# Emerging Microfluidic Tools for Functional Cellular Immunophenotyping: A New Potential Paradigm for Immune Status Characterization

**DOI:** 10.3389/fonc.2013.00098

**Published:** 2013-04-22

**Authors:** Weiqiang Chen, Nien-Tsu Huang, Xiang Li, Zeta Tak For Yu, Katsuo Kurabayashi, Jianping Fu

**Affiliations:** ^1^Integrated Biosystems and Biomechanics Laboratory, University of MichiganAnn Arbor, MI, USA; ^2^Department of Mechanical Engineering, University of MichiganAnn Arbor, MI, USA; ^3^Department of Electrical Engineering and Computer Science, University of MichiganAnn Arbor, MI, USA; ^4^Department of Biomedical Engineering, University of MichiganAnn Arbor, MI, USA

**Keywords:** immunophenotyping, microfluidics

## Abstract

Rapid, accurate, and quantitative characterization of immune status of patients is of utmost importance for disease diagnosis and prognosis, evaluating efficacy of immunotherapeutics and tailoring drug treatments. Immune status of patients is often dynamic and patient-specific, and such complex heterogeneity has made accurate, real-time measurements of patient immune status challenging in the clinical setting. Recent advances in microfluidics have demonstrated promising applications of the technology for immune monitoring with minimum sample requirements and rapid functional immunophenotyping capability. This review will highlight recent developments of microfluidic platforms that can perform rapid and accurate cellular functional assays on patient immune cells. We will also discuss the future potential of integrated microfluidics to perform rapid, accurate, and sensitive cellular functional assays at a single-cell resolution on different types or subpopulations of immune cells, to provide an unprecedented level of information depth on the distribution of immune cell functionalities. We envision that such microfluidic immunophenotyping tools will allow for comprehensive and systems-level immunomonitoring, unlocking the potential to transform experimental clinical immunology into an information-rich science.

## Introduction

The immune status of patients with infectious diseases and immune dysfunctions are dynamic and patient-specific, and such complex heterogeneity has made immunomodulatory therapies challenging in the clinic (Hotchkiss and Karl, 2003; Monneret et al., 2008). An accurate and real-time measurement of the immune status of patients is thus critical in disease diagnosis and prognosis, evaluating efficacy of immunotherapeutics, and tailoring drug treatments (Monneret et al., 2008). Functional cellular immunophenotyping, which measures the functional status of immune cells upon proliferation, cytolysis, and cytokine production, is arguably among the best methods to determine immune dysfunctions (Hotchkiss and Karl, 2003; Monneret et al., 2008; Lu et al., 2013). Immune cells in blood constitute a complex, heterogeneous mixture of multiple cell types including granulocytes, lymphocytes, and monocytes (Re and Strominger, 2004; Gordon and Taylor, 2005; Kaech and Wherry, 2007; O’Shea et al., 2008). The numbers, proportions, and cytolytic and cytokine production activities of leukocyte subsets change drastically in the presence of infections, malignancies, and autoimmune disorders (Revzin et al., 2012). As such, there is a significant need for reliable technologies that can perform rapid and accurate functional cellular immunophenotyping on patient immune cells and their subtypes to define and characterize the “immune phenotype” of patients.

Several approaches currently exist for assessment of the immune status of patients based on measuring cytokine production of immune cells. Enzyme-linked immunosorbent assay/spot (ELISA/ELISpot), for example, is a gold standard for quantifying cellular cytokine production (Cox et al., 2006; Cornell et al., 2012). ELISA/ELISpot has been commonly used for patients infected by malaria (Aidoo and Udhayakumar, 2000), HIV (Kern et al., 1999; Betts et al., 2000), and mycobacterium tuberculosis (Pathan et al., 2000) and monitoring the immune response of cancer patients undergoing immunotherapeutics (Janetzki et al., 2000; Lewis et al., 2000). However, ELISA/ELISpot usually requires numerous reagent manipulation processes that involve multiple staining, washing, blocking, and sample transfer steps, which are laborious and time-consuming. The complexity in implementing ELISA/ELISpot has been prohibitive for standardization and their utility in real-time clinical decision making. Further, ELISpot cannot quantify the amount of cytokine secretion, and it requires isolation and purification of desired subpopulations of immune cells prior to analysis, necessitating extensive sample preparation of blood specimens.

Functional cellular immunophenotyping can also be performed using intracellular cytokine staining (ICS) flow cytometry for single-cell cytokine production measurements with a high-throughput (>10^3^ cells/s) (Seder et al., 2008). However, ICS flow cytometry has so far only enabled detection of up to five cytokines, providing only a partial picture of the functional immune system. ICS flow cytometry also requires a large number of cells in suspension (>1 × 10^7^ cells in 1 mL solution) and is sample destructive, thus precluding downstream functional assays that require live cells. ICS flow cytometry has so far remained highly variable with regard to sample handling, reagents, instrument setup, and data analysis, thus standardization of ICS flow cytometry has been proved difficult if not impossible.

The limitations associated with conventional approaches to define the functional immune status of patients need to be fully addressed to realize rapid and accurate analysis of immune phenotype of patients, a key step that provides crucial information relating to staging, treatment choice, monitoring of efficacy, safety and dose adjustment of immunomodulation, as well as biological assessment of remission.

**Figure 1:**
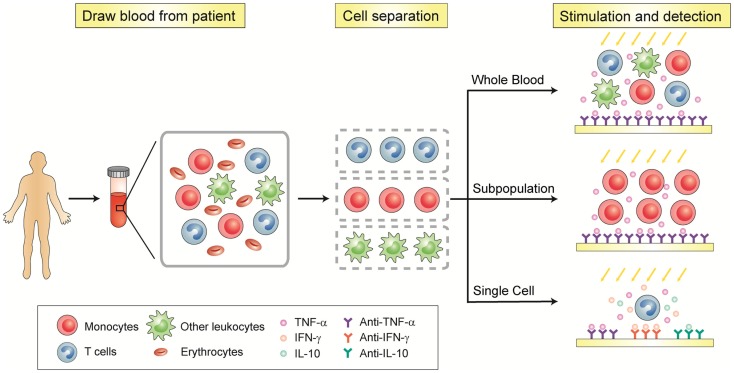
**Schematic of functional immunophenotyping of immune cells**.

Recent advances in integrated microfluidics have made possible miniaturization and integration of biosample preparative and analytical techniques on a single chip to enable rapid, sensitive, and multiplexed high-throughput on-chip cell-based assays. Some of these microfluidic tools have been demonstrated as promising immune monitoring technologies with cell trapping and analytic functionalities and a minimum sample requirement. This review will highlight the recent development of microfluidic platforms that can perform rapid and accurate whole-blood immunoassays of plasma components as well as functional cellular immunophenotyping assays for quantitative analysis of cytokine secretion properties of patient immune cells (Figure 1). We will particularly discuss the future potential of integrated microfluidics to perform rapid, accurate, and sensitive cellular functional assays at the single-cell resolution on immune cell subpopulations isolated directly from patient blood, and their potential to provide an unprecedented level of information depth on the distribution of immune cell functionalities on a patient-by-patient basis.

## Microfluidic Whole-Blood Immunoassays of Plasma Components

**Figure 2:**
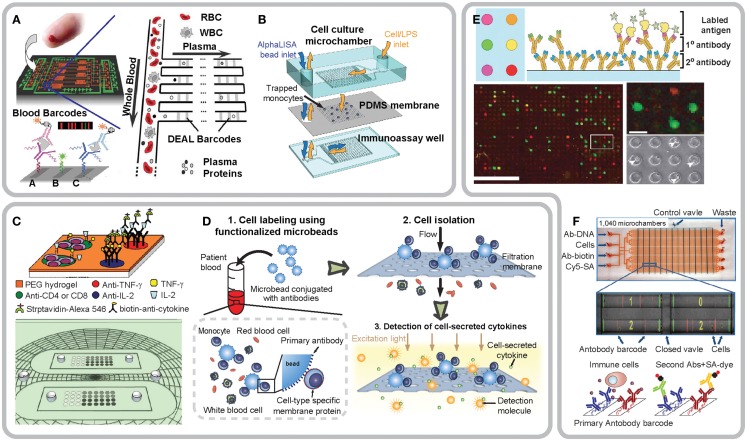
**Integrated microfluidic devices for functional immunophenotyping of immune cells in whole blood (A,B), subpopulations of immune cells (C,D) and single immune cells (E,F)**. **(A)** Design of the integrated blood barcode chip (IBBC). Adapted from Fan et al. (2008), Copyright © 2008, with permission from Nature Publishing Group. **(B)** Schematic of a multi-layered MIPA device consisting of a cell culture chamber, a PDMS microfiltration membrane (PMM), and an immunoassay chamber. Reproduced from Huang et al. (2012). **(C)** Conceptual design of an antibody-coated microarray for detection of cytokines secreted by CD4+ or CD8+ T-cells. Reproduced from Zhu et al. (2008) by permission of The Royal Society of Chemistry. **(D)** Schematic of isolation and immunophenotyping of subpopulations of immune cells from blood specimens by a combined use of both PMM and functionalized microbeads. Reproduced from Chen et al. (2013). **(E)** Working principle of the microengraving array for capture and immunomonitoring of single immune cells. Adapted from Love et al. (2006), Copyright © 2006, with permission from Nature Publishing Group. **(F)** Photograph and working principle of the single-cell barcode chip for polycytokine analysis of single immune cells. Adapted from Ma et al. (2011), Copyright © 2011, with permission from Nature Publishing Group.

Whole-blood immunoassay is a most commonly used method to examine patient immune status, which provides useful information for diagnosis (Boomer et al., 2011; Cornell et al., 2012), prognosis (Azizia et al., 2012), and deepening the biological understanding of immune and infectious diseases (Bernard et al., 2001; Hotchkiss and Opal, 2010). Conventional whole-blood immune tests are based on proteomic identification of biomarkers in blood, relying on antibody-based heterogeneous or homogeneous immunoassays (e.g., ELISA) to capture and recognize soluble biomarkers in blood specimens. Recently, to achieve rapid on-chip immunoassays with a minimum amount of blood, several microfluidic whole-blood immunoassay devices have been developed. A notable example is the integrated blood barcode chip reported by Fan et al. (2008) that can achieve on-chip plasma separation from microliter quantities of whole-blood and rapid *in situ* multiplexed protein biomarker measurements (Figure 2A). The marked performance of the blood barcode chip comes from its two integrated functional components: (1) a plasma-skimming channel that separates blood plasma based on the Zweifach–Fung effect; (2) a protein detection region using a patterned DNA-encoded antibody library (DEAL) barcode immobilized on the surface of the plasma-skimming channel. Specifically, the DEAL technology involves DNA-directed immobilization of antibodies to convert a prepatterned ssDNA barcode microarray into an antibody array, thus providing a powerful means for spatial encoding. The integrated blood barcode chip and its recent improvement reported by Wang et al. (2010) is capable of detecting picomolar concentrations of cancer biomarkers and more than 10 cytokines simultaneously from cancer patient blood.

## Microfluidic Whole-Blood Functional Immunoassays

In addition to proteomic analysis for soluble biomarkers in blood using microfluidic immunoassays, a recent exciting trend is to develop microfluidics-based cellular functional immune assays, which is arguably a more direct measurement of the functional status of immune cells. To achieve this, Huang et al. (2012) have recently developed a microfluidic immunophenotyping assay (MIPA) device for rapid and efficient on-chip isolation of peripheral blood mononuclear cells (PBMCs), their stimulation and cellular cytokine secretion measurements (Figure 2B). A key component of the MIPA device is a surface micromachined polydimethylsiloxane (*PDMS*) microfiltration membrane (PMM) for both isolation of PBMCs from blood and allowing cytokines secreted from lipopolysaccharide (LPS)-stimulated PBMCs to diffuse rapidly into a biosensing chamber for quantitative immunosensing. The MIPA device can achieve efficient on-chip cell isolation and enrichment from blood owing to the high porosity of the PMM as compared to existing polycarbonate filters (Vona et al., 2000; Hofman et al., 2011) or parylene-based micropore membranes (Zheng et al., 2011). For quantitative immunosensing, the MIPA device utilizes a commercially available homogeneous chemiluminescence technique, the “AlphaLISA,” which does not require any washing or blocking step, greatly shortening the total assay time and enhancing dynamic range for analyte detection. Owing to a miniaturized on-chip microfluidic environment, the MIPA device can achieve highly sensitive cellular immunophenotyping with 20-fold fewer cells as compared to standard whole-blood stimulation assay. The total assay time of the MIPA device using AlphaLISA is seven times faster than that of whole-blood stimulation assay using conventional ELISA.

Several microfluidics-based label-free, real-time detection techniques have also been developed recently for immunosensing. Development of real-time immunosensing techniques allows detailed examination of the temporal dynamics of cytokine secretion from immune cells, which may provide an informative and unique signature about the functional status of patient immune system (Revzin et al., 2012). The ability to assess dynamic cytokine secretion from immune cells, for example, can allow detection of the onset of the signaling process and study of intercellular communications via cytokine-mediated paracrine and autocrine signaling. Monitoring both the location and timing of cytokine secretion events among a heterogeneous population of individual immune cells can also determine which individual cells initiate the immune response and which cells are then activated by such initial immune response. In clinical diagnosis such as tuberculosis detection, pathogen biomarkers (e.g., pathogen-specific antibodies) are not yet available. As such, cytokine production by T-cells is commonly used as a diagnostic marker for tuberculosis. If detection of dynamic response of antigen-specific T cells becomes available, it will enable early pathogen detection before pathogen biomarkers are produced or the pathogen proliferates in the host.

A noteworthy microfluidic label-free immunodetection method has been recently reported by Stern et al. (2007) based on CMOS-compatible semiconducting nanowires for real-time measurements of antibodies and early signals responsible for T-cell activation. Another label-free biosensing technique reported by Endo et al. (2008) has applied immobilized antibodies and localized surface plasmon resonance (LSPR) to continuously monitor concentration levels of cytokines secreted from mouse thymus cells. The LSPR-based biosensor provides a promising platform with attractive advantages of real-time detection of cellular responses in a simplified experimental setup with a low sample volume requirement. Overall, label-free cellular immunophenotyping permits real-time quantifications of dynamic cytokine secretion, providing the unique functional signature of immune cells such as how fast and strong immune cells secrete cytokines in response to antigen stimulations.

## Microfluidic Immunophenotyping of Subpopulations of Immune Cells

Microfluidic whole-blood immunoassays measure the overall capacity of the whole population of leukocytes in blood to produce cytokines. Thus, microfluidic whole-blood immunoassays may not be informative enough to accurately reveal the immune status of patients, as in these “bulk” assays it is difficult to pinpoint the phenotype or real identity of reactive immune cells involved. Recently, there are great efforts from different research groups to integrate cell separation techniques into microfluidic immunoassay devices and systems to achieve cellular functional analysis on subpopulations of immune cells. Zhu et al. (2008) for example, have recently developed a microarray device uniformly coated with both T-cell capture antibodies (anti-CD4 and anti-CD8) and cytokine capture antibodies (anti-IFN-γ and anti-IL-2) on top of a poly(ethylene glycol) (PEG) hydrogel layer (Figure 2C). To enable capturing and positioning of single CD4+ and CD8+ T-cells, the antibody-coated microarray was covered with photolithographically patterned PEG hydrogel microwells on top of the antibody containing hydrogel layer. The antibody-coated microarray can directly process red blood cell (RBC) depleted human whole-blood samples for capture of individual CD4+ and CD8+ T-cells and subsequent functional examination of IFN-γ and IL-2 secretion from single T-cells.

The antibody-based microarray platform reported by Zhu et al. has simplified the sample preparation process and also reduced the required volume of blood specimens. Although immobilized antibodies offer a heightened cell isolation purity and cytokine measurement sensitivity, it still suffers from several limitations, including the need of multiple washing and blocking steps and the difficulty to achieve real-time dynamic cytokine secretion measurement. To address these limitations, the same research group has recently applied DNA and RNA-based aptamers as an alternative to antibodies and immobilized aptamers on an array of micropatterned gold electrodes (Zhu et al., 2009; Liu et al., 2012). The aptamers have been thiolated for assembly on gold and functionalized with a methylene blue redox reporter for electrochemical signal transduction and detection with gold electrodes. Instead of using fluorescence-based biosensing methods, the authors have successfully demonstrated electrochemical measurements to access dynamic cytokine secretion from human monocytes and T-cells with a detection sensitivity of ∼ng/mL (Zhu et al., 2009; Liu et al., 2012).

In addition to antibody- and aptamer-based immunophenotyping methods for subpopulations of immune cells, Chen et al. (2013) have recently developed an integrated microfluidic device employing a combined use of the PMM and antibody-conjugated polystyrene microbeads for isolation, purification, and functional immunophenotyping of subpopulation of immune cells directly from unprocessed blood specimens (Figure 2D). In their method, Chen et al. have first applied functionalized microbeads conjugated with monoclonal antibodies against specific cell surface proteins to label and enlarge targeted subpopulations of immune cells in blood specimens. After labeling using microbeads, blood specimen is introduced into the microfluidic device which contains the PMM. The cell/microbead conjugates are readily trapped and isolated on the PMM, whereas other untargeted blood cells unbound to microbeads can freely pass through the PMM. Following cell isolation, the AlphaLISA is applied for quantitative measurements of cytokine secretion from LPS-stimulated immune cells captured on the PMM.

## Microfluidics to Study Functional Heterogeneity of Single Immune Cells

Functional and phenotypic variation among individual single cells, or single-cell functional heterogeneity, is a common feature for hematopoietic cells including immune cells. Thus, quantitative functional analysis of immune cells down to a single-cell resolution is required for a precise assessment of patient immune status. Over the last decade, significant research efforts have been directed toward applying microfluidics for manipulation and functional analysis of single immune cells. One of the most notable example entails plating and stimulating single immune cells in an array of microfabricated wells, transferring soluble molecules secreted from immune cells onto a secondary solid surface coated with capture antibodies, and labeling captured molecules with fluorescently tagged proteins prior to subsequent optical detection. For example, Love et al. (2006) have pioneered the development of engraved microarrays made in PDMS using soft lithography to monitor cytokines secreted from single immune cells (Figure 2E). The engraved microarray consists of 25,000 microwells (50–100 μm in diameter), each of which confines single immune cells in a nanoliter volume. After individual immune cells trapped and stimulated, the engraved microarray can be flipped against an antigen- or secondary antibody-immobilized glass slide to capture primary antibodies secreted from cells. Compared to ELISpot, the engraved microarray enables a rapid (<12 h) and high-throughput (>10,000 individual cells) system for identification, recovery, and clonal expansion of single immune cells producing antigen-specific antibodies. More recently, studies from the same research group have demonstrated the capability of the engraved microarray for characterization of dynamic cytokine secretion from individual human T-cells after activation *ex vivo* (Han et al., 2012; Varadarajan et al., 2012).

In addition to the microengraving method, Jin et al. (2009) have recently independently developed a functional immunosensing technique called “immunospot array assay on a chip” (or ISAAC) to detect production of monoclonal antibodies by immune cells. The ISAAC method offers a rapid and high-throughput system for screening and analysis of antigen-specific antibody-secreting cells (ASCs) on a single-cell basis. Similar to the microengraving assay, the ISAAC also includes an array of microwells for trapping of single live immune cells. The top surface of ISAAC is functionalized with antibodies against immunoglobulin, and antibodies secreted by individual ASCs trapped in the wells are captured and bound to the device surface around the well. The ISAAC method is useful for detecting ASCs in response to different antigens as well as for selection of ASCs secreting high-affinity antibodies. Although both the microengraving and ISAAC methods have used a high-density array of microwells to trap and isolate single immune cells, the two methods utilize different detection techniques. Fundamentally, the microengraving method pioneered by Love et al. (2006) is based on ELISA, whereas the ISAAC is based on ELISpot (Jin et al., 2009).

Ma et al. (2011) have recently applied the single-cell barcode chip for high-content assessment of the functional heterogeneity of antigen-specific T-cells (Figure 2F). The single-cell barcode chip consists of 1,040 microchambers with a nanoliter volume, and each microchamber can trap single or a small number of immune cells. On the bottom surface of each microchamber, a spatially encoded antibody barcode array is pre-printed to capture cytokines secreted from immune cells trapped in the microchamber. Protein concentrations are measured with immunosandwich assays from the spatially encoded antibody barcode. A full barcode from each microchamber represents a complete panel of multiple cytokine species produced by a single immune cell (or a few cells). The single-cell barcode chip permits highly multiplexed (more than 10 proteins) on-chip detection of a few thousand proteins or less from thousands of immune cells simultaneously. The single-cell barcode chip reported by Ma et al. represents an exciting and informative microfluidic single-cell immunophenotyping tool for analyzing functional signatures of immune cells with high sensitivity, throughput and multiplicity, and a small sample size requirement.

All the microfluidic devices and systems discussed in this section provide a promising potential for high-throughput study of the functional heterogeneity of single immune cells. However, one critical issue common with these approaches is that they require off-chip isolation and purification of target cells from whole-blood prior to on-chip analysis. As such, there is still an unmet need for a highly integrated microfluidic technology platform for efficient isolation and informative systems-level cellular characterization of immune cells down to the single-cell level and using unprocessed or minimally processed blood samples.

## Conclusion

Developing reliable, multiplexed biosensing techniques that permit simultaneous characterization of the functional status of different subpopulations of immune cells at a single-cell resolution is an exciting emerging concept. This concept holds a great promise for unraveling pathogenesis as well as for translating newly available therapeutic options into optimal personalized treatments. Continued progress in many fields ranging from fundamental immunology studies and clinical discoveries to patient managements critically hinges on the availability of such immune monitoring systems. Recent exciting developments in microfluidic technology have provided promising tools for functional cellular immunophenotyping of blood specimens. These microfluidic immunophenotyping techniques can potentially provide an unprecedented level of information depth on the distribution of immune cell functionalities. We envision that such microfluidic immunophenotyping tools will allow comprehensive and systems-level immunomonitoring in the future, thus unlocking the potential to transform experimental clinical immunology into an information-rich science.

## Conflict of Interest Statement

The authors declare that the research was conducted in the absence of any commercial or financial relationships that could be construed as a potential conflict of interest.

## References

[B1] Aidoo M. Udhayakumar V. (2000). Field studies of cytotoxic T lymphocytes in malaria infections: implications for malaria vaccine development. Parasitol. Today (Regul. Ed.) 16, 50–56 10.1016/S0169-4758(99)01592-6 10652487

[B2] Azizia M. Lloyd J. Allen M. Klein N. Peebles D. (2012). Immune status in very preterm neonates. Pediatrics 129, e967–e974 10.1542/peds.2011-1579 22451711

[B3] Bernard G. R. Vincent J. L. Laterre P. F. LaRosa S. P. Dhainaut J. F. Lopez-Rodriguez A. (2001). Efficacy and safety of recombinant human activated protein c for severe sepsis. N. Engl. J. Med. 344, 699–709 10.1056/NEJM200103083441001 11236773

[B4] Betts M. R. Casazza J. P. Patterson B. A. Waldrop S. Trigona W. Fu T. M. (2000). Putative immunodominant human immunodeficiency virus-specific cd8(+) T-cell responses cannot be predicted by major histocompatibility complex class I haplotype. J. Virol. 74, 9144–9151 10.1128/JVI.74.19.9144-9151.2000 10982361 PMC102113

[B5] Boomer J. S. To K. Chang K. C. Takasu O. Osborne D. F. Walton A. H. (2011). Immunosuppression in patients who die of sepsis and multiple organ failure. J. Am. Med. Assoc. 306, 2594–2605 10.1001/jama.2011.1829 PMC3361243 22187279

[B6] Chen W. Huang N. T. Oh B. Lam R. H. Fan R. Cornell T. T. (2013). Surface-micromachined microfiltration membranes for efficient isolation and functional immunophenotyping of subpopulations of immune cells. Adv. Healthc. Mater. 10.1002/adhm.201200378 PMC4459734 23335389

[B7] Cornell T. T. Sun L. Hall M. W. Gurney J. G. Ashbrook M. J. Ohye R. G. (2012). Clinical implications and molecular mechanisms of immunoparalysis after cardiopulmonary bypass. J. Thorac. Cardiovasc. Surg. 143, 1160.e1–1166.e1 10.1016/j.jtcvs.2011.09.011 21996297 PMC3262077

[B8] Cox J. H. Ferrari G. Janetzki S. (2006). Measurement of cytokine release at the single cell level using the ELISpot assay. Methods 38, 274–282 10.1016/j.ymeth.2005.11.006 16473524

[B9] Endo T. Yamamura S. Kerman K. Tamiya E. (2008). Label-free cell-based assay using localized surface plasmon resonance biosensor. Anal. Chim. Acta 614, 182–189 10.1016/j.aca.2008.03.022 18420049

[B10] Fan R. Vermesh O. Srivastava A. Yen B. K. Qin L. Ahmad H. (2008). Integrated barcode chips for rapid, multiplexed analysis of proteins in microliter quantities of blood. Nat. Biotechnol. 26, 1373–1378 10.1038/nbt.1507 19029914 PMC2775523

[B11] Gordon S. Taylor P. R. (2005). Monocyte and macrophage heterogeneity. Nat. Rev. Immunol. 5, 953–964 10.1038/nri1733 16322748

[B12] Han Q. Bagheri N. Bradshaw E. M. Hafler D. A. Lauffenburger D. A. Love J. C. (2012). Polyfunctional responses by human T cells result from sequential release of cytokines. Proc. Natl. Acad. Sci. U.S.A. 109, 1607–1612 10.1073/pnas.1117194109 22160692 PMC3277116

[B13] Hofman V. J. Ilie M. I. Bonnetaud C. Selva E. Long E. Molina T. (2011). Cytopathologic detection of circulating tumor cells using the isolation by size of epithelial tumor cell method: promises and pitfalls. Am. J. Clin. Pathol. 135, 146–156 10.1309/AJCP9X8OZBEIQVVI 21173137

[B14] Hotchkiss R. S. Karl I. E. (2003). The pathophysiology and treatment of sepsis. N. Engl. J. Med. 348, 138–150 10.1056/NEJMra021333 12519925

[B15] Hotchkiss R. S. Opal S. (2010). Immunotherapy for sepsis – a new approach against an ancient foe. N. Engl. J. Med. 363, 87–89 10.1056/NEJMcibr1004371 20592301 PMC4136660

[B16] Huang N. T. Chen W. Oh B. R. Cornell T. T. Shanley T. P. Fu J. (2012). An integrated microfluidic platform for in situ cellular cytokine secretion immunophenotyping. Lab. Chip 12, 4093–4101 10.1039/c2lc21142d 22892681 PMC3508001

[B17] Janetzki S. Palla D. Rosenhauer V. Lochs H. Lewis J. J. Srivastava P. K. (2000). Immunization of cancer patients with autologous cancer-derived heat shock protein gp96 preparations: a pilot study. Int. J. Cancer 88, 232–238 10.1002/1097-0215(20001015)88:2<232::AID-IJC14>3.0.CO;2-8 11004674

[B18] Jin A. Ozawa T. Tajiri K. Obata T. Kondo S. Kinoshita K. (2009). A rapid and efficient single-cell manipulation method for screening antigen-specific antibody-secreting cells from human peripheral blood. Nat. Med. 15, 1088–1092 10.1038/nm.1966 19684583

[B19] Kaech S. M. Wherry E. J. (2007). Heterogeneity and cell-fate decisions in effector and memory cd8+ T cell differentiation during viral infection. Immunity 27, 393–405 10.1016/j.immuni.2007.08.007 17892848 PMC3431921

[B20] Kern F. Surel I. P. Faulhaber N. Frommel C. Schneider-Mergener J. Schonemann C. (1999). Target structures of the cd8(+)-T-cell response to human cytomegalovirus: the 72-kilodalton major immediate-early protein revisited. J. Virol. 73, 8179–8184 10482568 10.1128/jvi.73.10.8179-8184.1999 PMC112835

[B21] Lewis J. J. Janetzki S. Schaed S. Panageas K. S. Wang S. Williams L. (2000). Evaluation of cd8(+) T-cell frequencies by the ELISpot assay in healthy individuals and in patients with metastatic melanoma immunized with tyrosinase peptide. Int. J. Cancer 87, 391–398 10.1002/1097-0215(20000801)87:3<391::AID-IJC13>3.0.CO;2-K 10897045

[B22] Liu Y. Kwa T. Revzin A. (2012). Simultaneous detection of cell-secreted TNF-alpha and IFN-gamma using micropatterned aptamer-modified electrodes. Biomaterials 33, 7347–7355 10.1016/j.biomaterials.2012.08.010 22809645 PMC3863689

[B23] Love J. C. Ronan J. L. Grotenbreg G. M. van der Veen A. G. Ploegh H. L. (2006). A microengraving method for rapid selection of single cells producing antigen-specific antibodies. Nat. Biotechnol. 24, 703–707 10.1038/nbt1210 16699501

[B24] Lu Y. Chen J. J. Mu L. Xue Q. Wu Y. Wu P. H. (2013). High-throughput secretomic analysis of single cells to assess functional cellular heterogeneity. Anal. Chem. 85, 2548–2556 10.1021/ac4004248 23339603 PMC3589817

[B25] Ma C. Fan R. Ahmad H. Shi Q. Comin-Anduix B. Chodon T. (2011). A clinical microchip for evaluation of single immune cells reveals high functional heterogeneity in phenotypically similar T cells. Nat. Med. 17, 738–743 10.1038/nm.2375 21602800 PMC3681612

[B26] Monneret G. Venet F. Pachot A. Lepape A. (2008). Monitoring immune dysfunctions in the septic patient: a new skin for the old ceremony. Mol. Med. 14, 64–78 10.2119/2007-00102.Monneret 18026569 PMC2078557

[B27] O’Shea J. J. Hunter C. A. Germain R. N. (2008). T cell heterogeneity: firmly fixed, predominantly plastic or merely malleable? Nat. Immunol. 9, 450–453 10.1038/ni0508-450 18425095

[B28] Pathan A. A. Wilkinson K. A. Wilkinson R. J. Latif M. McShane H. Pasvol G. (2000). High frequencies of circulating IFN-gamma-secreting cd8 cytotoxic T cells specific for a novel MHC class I-restricted *Mycobacterium tuberculosis* epitope in *M. Tuberculosis*-infected subjects without disease. Eur. J. Immunol. 30, 2713–2721 10.1002/1521-4141(200009)30:9<2713::AID-IMMU2713>3.0.CO;2-4 11009107

[B29] Re F. Strominger J. L. (2004). Heterogeneity of TLR-induced responses in dendritic cells: from innate to adaptive immunity. Immunobiology 209, 191–198 10.1016/j.imbio.2004.03.005 15481153

[B30] Revzin A. Maverakis E. Chang H. C. (2012). Biosensors for immune cell analysis – a perspective. Biomicrofluidics 6, 21301–21313 10.1063/1.4706845 22655003 PMC3360707

[B31] Seder R. A. Darrah P. A. Roederer M. (2008). T-cell quality in memory and protection: implications for vaccine design. Nat. Rev. Immunol. 8, 247–258 10.1038/nri2274 18323851

[B32] Stern E. Klemic J. F. Routenberg D. A. Wyrembak P. N. Turner-Evans D. B. Hamilton A. D. (2007). Label-free immunodetection with cmos-compatible semiconducting nanowires. Nature 445, 519–522 10.1038/nature05498 17268465

[B33] Varadarajan N. Kwon D. S. Law K. M. Ogunniyi A. O. Anahtar M. N. Richter J. M. (2012). Rapid, efficient functional characterization and recovery of HIV-specific human cd8+ T cells using microengraving. Proc. Natl. Acad. Sci. U.S.A. 109, 3885–3890 10.1073/pnas.1111205109 22355106 PMC3309713

[B34] Vona G. Sabile A. Louha M. Sitruk V. Romana S. Schutze K. (2000). Isolation by size of epithelial tumor cells: a new method for the immunomorphological and molecular characterization of circulating tumor cells. Am. J. Pathol. 156, 57–63 10.1016/S0002-9440(10)64706-2 10623654 PMC1868645

[B35] Wang J. Ahmad H. Ma C. Shi Q. Vermesh O. Vermesh U. (2010). A self-powered, one-step chip for rapid, quantitative and multiplexed detection of proteins from pinpricks of whole blood. Lab. Chip 10, 3157–3162 10.1039/c003264f 20924527 PMC3651856

[B36] Zheng S. Lin H. K. Lu B. Williams A. Datar R. Cote R. J. (2011). 3D microfilter device for viable circulating tumor cell (ctc) enrichment from blood. Biomed. Microdevices 13, 203–213 10.1007/s10544-010-9485-3 20978853 PMC3809998

[B37] Zhu H. Stybayeva G. Macal M. Ramanculov E. George M. D. Dandekar S. (2008). A microdevice for multiplexed detection of T-cell-secreted cytokines. Lab. Chip 8, 2197–2205 10.1039/b810244a 19023487

[B38] Zhu H. Stybayeva G. Silangcruz J. Yan J. Ramanculov E. Dandekar S. (2009). Detecting cytokine release from single T-cells. Anal. Chem. 81, 8150–8156 10.1021/ac802203r 19739655 PMC2755614

